# Global gateways as telecoupled human and natural systems: The emerging case of the Bering Strait

**DOI:** 10.1007/s13280-023-01835-2

**Published:** 2023-03-28

**Authors:** Sydney Waloven, Kelly Kapsar, Tobias Schwoerer, Matthew Berman, Jennifer I. Schmidt, Andrés Viña, Jianguo Liu

**Affiliations:** 1 Department of Fisheries and Wildlife, Center for Systems Integration & Sustainability, Michigan State University, 115 Manly Miles Building, 1405 S. Harrison Rd., East Lansing, MI 48823 USA; 2 International Arctic Research Center, University of Alaska Fairbanks, 2160 Koyukuk Drive, PO Box 757340, Fairbanks, AK 99775-7340 USA; 3 Institute of Social and Economic Research, University of Alaska Anchorage, 3211 Providence Drive, Anchorage, AK 99508 USA

**Keywords:** Arctic, Coupled human and natural system, Natural resource development, Shipping, Telecoupling, Tourism

## Abstract

Numerous narrow marine passages around the world serve as essential gateways for the transportation of goods, the movement of people, and the migration of fish and wildlife. These global gateways facilitate human–nature interactions across distant regions. The socioeconomic and environmental interactions among distant coupled human and natural systems affect the sustainability of global gateways in complex ways. However, the assessment and analysis of global gateways are scattered and fragmented. To fill this knowledge gap, we frame global gateways as telecoupled human and natural systems using an emerging global gateway, the Bering Strait, as a demonstration. We examine how three telecoupling processes (tourism, vessel traffic, and natural resource development) impact and are impacted by the coupled human and natural system of the Bering Strait Region. Given that global gateways share many similarities, our analysis of the Bering Strait Region provides a foundation for the assessment of other telecoupled global gateways.

## Introduction

Narrow marine passages around the world are essential corridors for the transportation of goods and the migration of wildlife. With over 80% of the world’s trade transported by marine shipping (United Nations 2017), these passages, hereafter global gateways, serve as key thoroughfares that concentrate vessel traffic and improve the efficiency of the transportation of goods around the world. Examples of global gateways include the Bosporus Strait, the English Channel, the Strait of Gibraltar, the Strait of Magellan, the Panama Canal, the Singapore Strait, and the Suez Canal (Table 1). While global gateways vary in size, function, and degree of anthropogenic and ecological activity, they share many similarities. Besides their common functions as corridors, global gateways function as coupled human and natural systems (CHANS), in which humans and natural components interact (Liu et al. 2007). Many global gateways experience human activities such as fishing, tourism (e.g., cruise ships), subsistence hunting, and oil and gas development. Further, many global gateways, such as Panama Canal and Strait of Magellan, are located within the homelands of Indigenous Peoples with distinct cultures and practices. Still other global gateways serve as key habitat and migratory corridors for marine wildlife, concentrating the potential for human-wildlife conflicts (e.g., Bering Strait, Strait of Magellan).

**Table 1:** Global gateways and their associated trade routes

Global gateway	Connected waterbodies	Trade routes
Bering Strait	Pacific Ocean, Arctic Ocean	Asia ←→ Europe, N. America West Coast
Bosporus Strait	Sea of Marmara, Black Sea	Asia ←→ Europe
English channel	Atlantic Ocean, North Sea	Southern England ←→ France
Strait of Gibraltar	Atlantic Ocean, Mediterranean Sea	Southern Europe, Northern Africa ←→ Western Asia
Strait of Magellan	Pacific Ocean, Atlantic Ocean	Asia ←→ Europe S. America West Coast ←→ S. America East Coast
Panama canal	Pacific Ocean, Atlantic Ocean	N. America and Central America East Coast ←→ N. America West Coast
Singapore Strait	Indian Ocean, South China Sea	Middle East ←→ Southeast Asia
Suez canal	Mediterranean Sea, Red Sea	Asia ←→ Europe

The movement of people and goods into, out of, or through global gateways results in several overlapping telecoupling processes such as tourism and trade (Hull and Liu 2018). Telecouplings occur when flows of people, goods, materials, information, or energy pass between distant CHANS (or social-ecological systems; (Liu et al. 2007)). According to the integrated framework of telecoupling, sending systems transmit flows, while receiving systems obtain them. Systems that are affected by the flow between sending and receiving systems are called spillover systems (Liu et al. 2013, 2018; Zhao et al. 2020). By serving as the most direct route between major ports, global gateways may increase the speed and efficiency of the transportation of goods around the world. Furthermore, some global gateways, such as the Singapore Strait, have ports that benefit economically from the trade that passes through them. However, large amounts of ship traffic can have detrimental effects on surrounding air quality (Aliabadi et al. 2015), and also pose numerous risks to marine and coastal environments, including oil spills, noise pollution, and ship collisions with marine mammals (AMSA 2009; Allen 2014; Huntington et al. 2015; Helle et al. 2020). The reduction of human-wildlife conflicts within marine gateways is essential for maintaining the resilience of coupled human and natural systems to a changing climate, environment, and to increasing trade (Nyhus 2016). Identifying and quantifying these complexities, such as the economic benefits of trade or environmental impacts, is an essential step toward understanding and developing strategies to effectively manage the sustainability of global gateways.

There have been many interdisciplinary CHANS studies analyzing human–environment interactions in the Arctic, as well as disciplinary studies focused on isolated processes at community or regional scales (Kapsar et al. 2022). Yet, analyzing processes this way may miss the intricacies and interacting effects of multiple telecouplings. Additionally, the synthesis of research findings of shipping corridors as CHANS is largely fragmented and scattered. Applying the telecoupling framework to conceptualize global gateways as telecoupled human and natural systems can better integrate results from specialized or localized analyses (Liu et al. 2013). Furthermore, these results can better reflect how disturbances in distant systems can compound with other processes and their effects locally. To better understand the ways that multiple telecouplings affect the social-ecological sustainability of global gateways, we use the Bering Strait as an example.

**Fig. 1:**
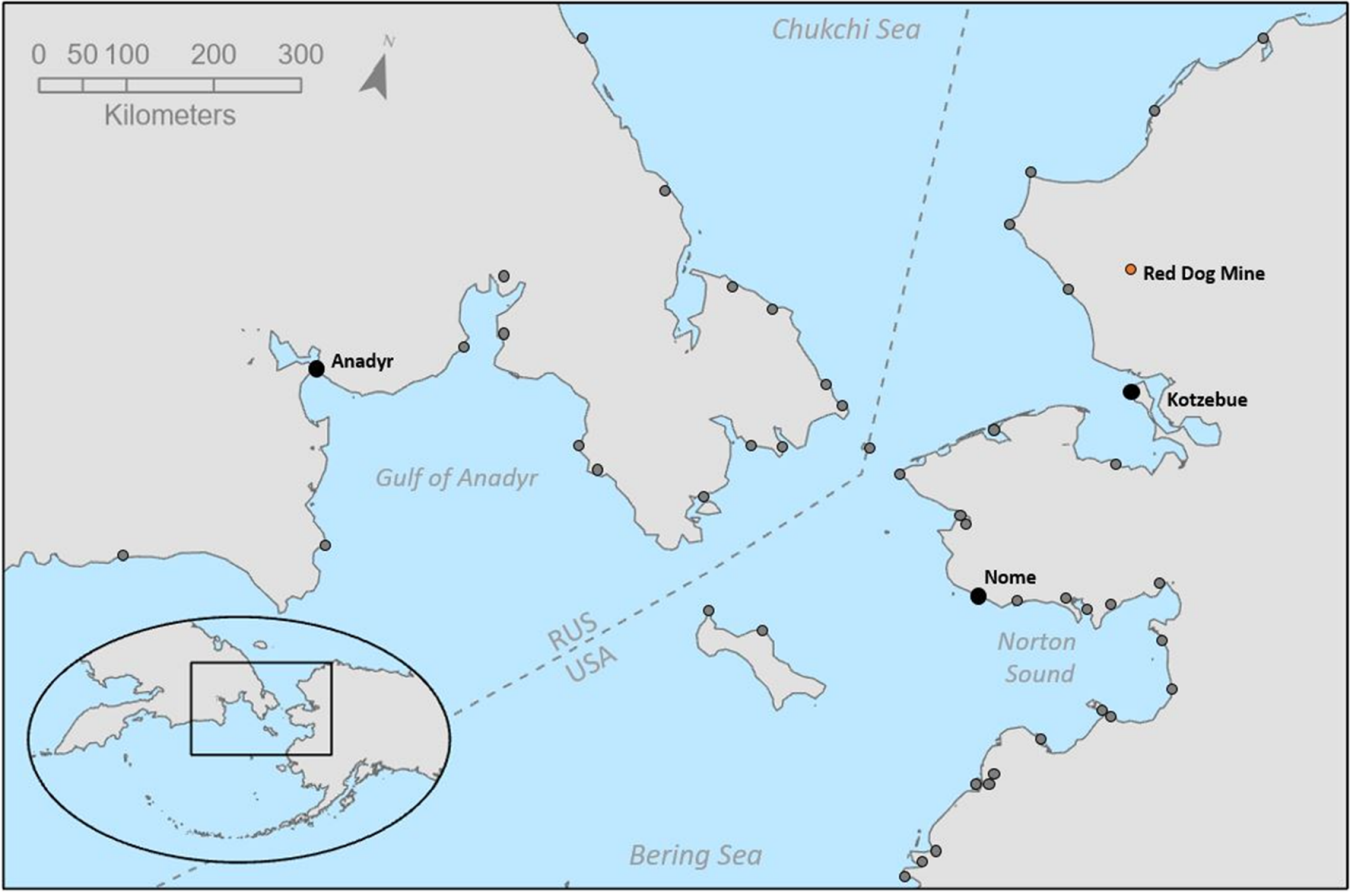
Map of the Bering Strait Region, featuring the Red Dog Mine and hub communities of Anadyr, Kotzebue, and Nome. Gray dots indicate additional coastal communities of the BSR

We begin our analysis by characterizing the geographic and socio-ecological importance of the Bering Strait, based on existing literature. To fully encompass the socio-ecological significance of this emerging gateway, we consider not just the Bering Strait itself, but the broader Bering Strait Region (BSR) which incorporates the gateway and its surrounding area, including multiple coastal and primarily Indigenous communities as well as diverse marine ecosystems (Fig. 1). We then examine the many telecouplings that link the BSR to distant systems, including the effects they currently or could possibly have on the future management of human-wildlife interactions and sustainability of wildlife habitats within the BSR. Finally, we evaluate current governance structures that influence the strength and distribution of telecouplings within the BSR. The influence of government policies highlights the need for policies that reduce the potential for vessel-wildlife conflicts, environmental degradation, safety risks for local communities, and other impacts on the coupled human and natural system. The understudied interactions and processes of one place and its potential to compromise the sustainability of another region may have important and unintended consequences for humans and wildlife in the Arctic. Therefore, we conclude with an examination of knowledge gaps regarding global gateways and suggestions of future directions for research.

## The Bering Strait as an emerging global gateway

Located in the North Pacific, the Bering Strait is an approximately 80 km wide passage between the Pacific and Arctic Oceans that divides the Russian Federation’s Chukotka Peninsula and Alaska’s Seward Peninsula (Fig. 1) (Allen 2014). The maritime portions of the BSR include the southern Chukchi Sea, the northern Bering Sea, and the neighboring coastal areas of the United States and the Russian Federation (Berkman et al. 2016). The BSR is located within the Arctic sub-Arctic ecotone (Fig. 1), which supports a rich species diversity, including a high abundance of seabirds and marine mammals (Huntington et al. 2015; Hauser et al. 2021; Vynne et al. 2021). Several major ocean currents flow through the BSR, carrying nutrient-rich water that nourishes phytoplankton, zooplankton, and benthic productivity (Hartsig et al. 2012).

The BSR experiences drastic seasonal changes brought about by the growth and retreat of sea ice (Barnhart et al. 2016; Alaska Ocean Observing System and International Arctic Research Center 2021). Each winter, as temperatures drop, sea ice extends southward from the Arctic Ocean, through the Bering Strait and into the Bering Sea. Historically, sea ice covered the region continuously from approximately the end of October to May (Barnhart et al. 2016), when warmer temperatures caused the annual break up and northward retreat of the ice edge. However, during the winter of 2020–2021, sea ice did not cover this region until mid-December, a situation that has become more common in recent years (Alaska Ocean Observing System and International Arctic Research Center 2021). This growing open-water period is already influencing regional weather and climate regimes as well as storm systems and southerly winds and is expected to perpetuate into the future as these interannual warm events become more frequent. Coastal erosion due, in part, to the absence of the buffering effects of sea ice has already had devastating consequences for local communities, such as the community of Shishmaref, which is facing the need to relocate entirely (Marino 2012).

The dynamics of sea ice shape the movements of humans and wildlife in the BSR, creating a dynamic CHANS (Liu et al. 2007; Fidel et al. 2014; Mahoney et al. 2021). The habitat of the BSR supports many marine mammals during feeding, pupping, breeding, resting, and migrating (Hartsig et al. 2012; Hauser et al. 2021). Notable pinniped species for their subsistence and cultural value include the Pacific walrus (*Odobenus rosmarus divergens*), the bearded seal (*Erignathus barbatus*), and the spotted seal (*Phoca largha*) (Hartsig et al. 2012; Smith et al. 2017). Other species, including bowhead whales (*Balaena mysticetus*) and polar bears (*Ursus maritimus*), follow the ice edge southward each winter and retreat to the north in the summer. These movements further shape the seasonal wild food harvest patterns and ensure the food security of Indigenous subsistence hunters whose wellbeing is intimately interconnected with a functional Arctic ecosystem (Inuit Circumpolar Council 2014; Arctic Council 2021).

Several Indigenous Peoples call the BSR home, speaking a variety of Indigenous languages such as Iñupiaq, Chukot, Central Yup’ik, and Siberian Yupik (Krauss 1974; Hartsig et al. 2012). Cultural diversity contributes to the activity and significance of this important region (Meek et al. 2008; Raymond-Yakoubian et al. 2017; Vynne et al. 2021). Indigenous communities in the BSR rely on marine resources for diet fulfillment, cultural identity, and social cohesion (Larsen Tempel et al. 2021). In addition to providing nutritious food, subsistence harvesting is an invaluable cultural practice for many Indigenous communities in the BSR (Quakenbush et al. 2016). Unconsumed portions of harvested animals are frequently used for the creation of authentic handicrafts and clothing (Larsen Tempel et al. 2021), and contribute to an ongoing mixed cash and subsistence economy (BurnSilver et al. 2016). Subsistence harvesters in this region have noted that species of subsistence interest (e.g., walruses, seals) are becoming increasingly difficult to access as a result of sea ice loss and its distributional shifts (Huntington et al. 2020; Hauser et al. 2021; Larsen Tempel et al. 2021). More frequent open water and thinned sea ice is also leading to unpredictable regional weather patterns and impacting climate regimes (Ballinger and Overland 2022). Without the protection of sea ice, this open water is exposing subsistence harvesters to greater risks during hunting such as more difficult and often longer travel across rougher seas with greater exposure to larger ocean swells (Larsen Tempel et al. 2021).

The significance of the BSR stems from its role as a natural system gateway for the migration of wildlife through its profoundly productive waters and simultaneous support of Indigenous livelihoods. As climate change and economic development further connect the BSR to distant systems, the sustainability of the BSR is increasingly impacted by external forces. In the following section, we discuss these connections and their implications for the sustainability of the coupled human and natural system of the BSR in further detail.

## Telecouplings between the Bering Strait Region and the rest of the world

Global gateways act as geographic ‘bottlenecks’, concentrating human activities as well as geophysical and ecological processes. As the only access point between the Pacific and Arctic Oceans, the Bering Strait serves as an important seasonal thoroughfare for many types of shipping related activities. The BSR is also tied to many distant systems through flows of resources, information, and people from around the world that travel into, out of, and through the BSR. The multi-directional movement of flows makes the BSR a sending, receiving, or spillover system, depending on the direction of the flow being analyzed. For example, the BSR acts as a receiving system of external shipping vessels bringing resources to remote communities. As the BSR distributes natural resources extracted locally (e.g., oil, minerals, fish) to outside markets, it acts as a sending system. Lastly, the BSR undergoes spillover effects from other sending and receiving systems that originate in distant regions, such as the Yamal Peninsula of Russia that utilizes the Bering Strait to transport resources (e.g., liquefied natural gas (LNG)) to distant markets (Parks et al. 2019).

**Fig. 2:**
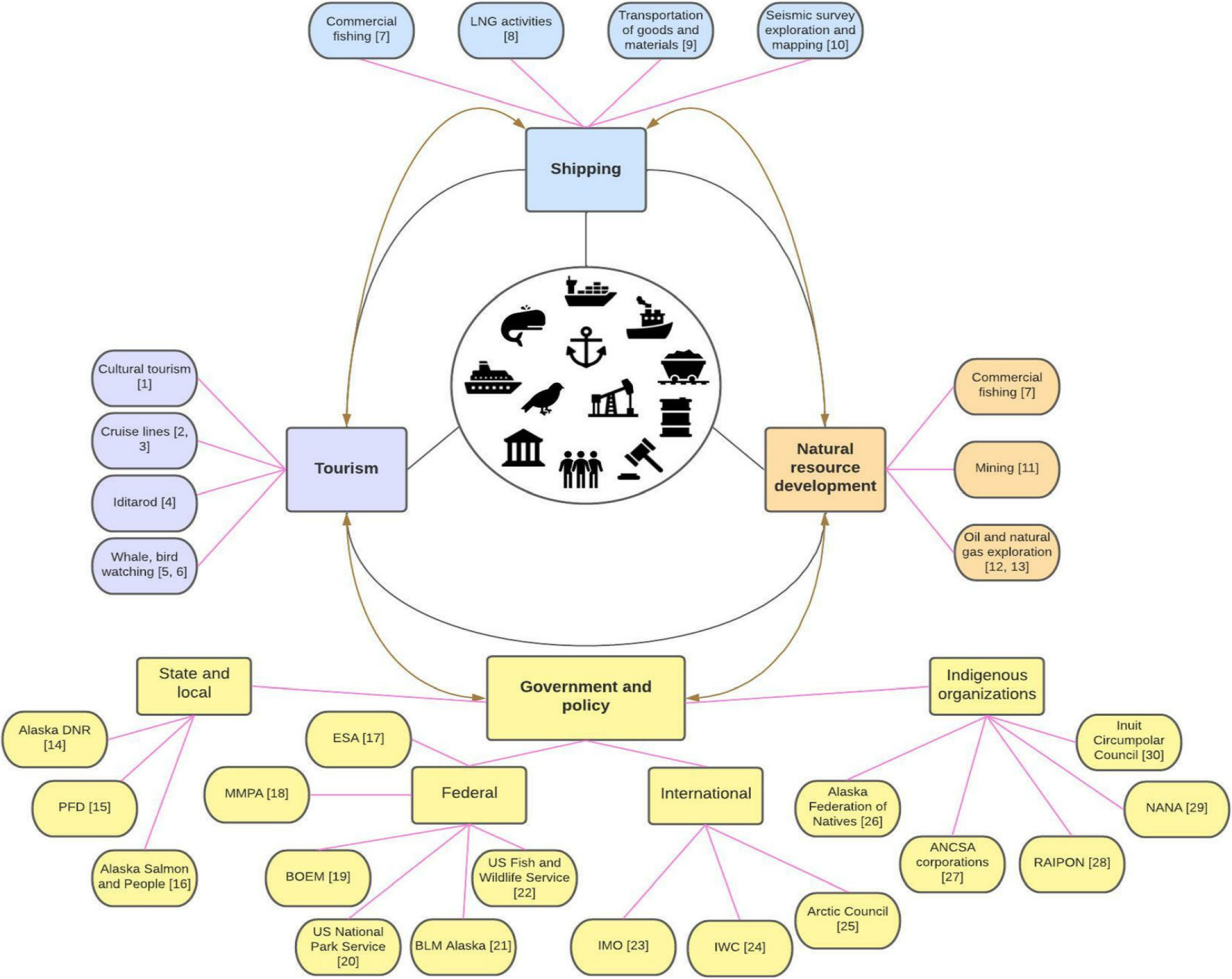
Bering Strait telecoupling examples and their components. Black lines represent connections to telecouplings. Pink lines represent connections to components or activity examples. Brown arrows represent feedbacks of government and policy, either influencing or responding to telecouplings between this region and other parts of the world. [1] (Liu and Lu 2014) [2] (Wang et al. 2020) [3] (Cajaiba-Santana et al. 2020) [4] (Blakeson et al. 2022) [5] (Heckel 2001) [6] https://www.adfg.alaska.gov/index.cfm?adfg=birdviewing.hotspots [7] (Carlson et al. 2020) [8] (Boylan 2021) [9] (United Nations 2017) [10] (Hartsig et al. 2012) [11] (Tolvanen et al. 2019) [12] (Bird et al. 2008) [13] (Nong et al. 2018) [14] *Alaska Department of Natural Resources* http://dnr.alaska.gov/ [15] *Permanent Fund Dividend* https://pfd.alaska.gov/ [16] (Carothers et al. 2021) [17] (Endangered Species Act 16 U.S.C. § 1531–1544 (1973)) [18] (Marine Mammal Protection Act, 16 U.S.C. § 1361–1407 (1994)) [19] *Bureau of Ocean Management* https://www.boem.gov/regions/alaska-ocs-region [20] https://www.nps.gov/state/ak/index.htm [21] *Bureau of Land Management Alaska* https://www.blm.gov/alaska [22] https://fws.gov/about/region/alaska [23] *International Maritime Organization* (Berkman et al. 2016) [24] *International Whaling Commission* https://iwc.int/en/ [25] https://arctic-council.org/ [26] www.nativefederation.org [27] *Alaska Native Claims Settlement Act* https://ancsaregional.com/ [28] *Russian Association of Indigenous Peoples of the North* (Kaczynski 2013) [29] (Due Kadenic 2015) [30] https://www.inuitcircumpolar.com/

Below, we discuss three main telecouplings affecting the marine systems of the BSR: vessel traffic, natural resource development, and tourism. Components of these telecouplings are summarized in Fig. 2. Additionally, we discuss actors involved in the governance of the BSR as well as relevant policies that influence the strength of telecouplings (Fig. 2).

### Vessel traffic

Vessel traffic in the Bering Strait Region serves a wide variety of purposes. First and foremost, vessel traffic is an essential service which provides resources, such as fuel, to remote communities (particularly on the Alaskan side of the BSR) that are not connected by roads. Tug and barge traffic also provides communities with other essential supplies, including building materials, food, and vehicles. In this context, the BSR serves as a receiving system for materials from distant systems with residents subject to global economic processes as reflected in the price of these goods.

The BSR also serves as a key thoroughfare providing access to the two main Arctic shipping routes: the Northern Sea Route (NSR) north of Asia and the Northwest Passage (NWP) through Canada. The majority of traffic along these routes is destinational, primarily taking resources into and out of the Arctic rather than passing through the Arctic in route to non-Arctic destinations (i.e., transit shipping) (AMSA 2009; PAME 2020). For both geographical and political reasons, vessel traffic along the Northern Sea Route is likely to grow faster than the Northwest Passage. The use of the NSR is influenced by heavily interconnected climatic, geographic, and legal factors which must all be considered when using this route in the future. For example, global geopolitical relationships with Russia have changed dramatically since February of 2022 when Russia invaded Ukraine (Liu et al. 2022). This event has had cascading consequences for the Arctic, including a cessation of Arctic Council activities (US Department of State 2022). Long-term impacts on vessel traffic along the Northern Sea Route could occur depending on the shifting nature of geopolitical relationships between Russia and Asia as well as Russia and western countries like the USA and those in Europe. However, the exact nature of these impacts remains to be seen.

There are also many factors and risks deeming the development of the NSR as unprofitable and unsafe, such as higher capital cost of ice-enforced ships and winterization of equipment, variability of sea ice and extreme weather, remoteness, limited satellite communications, price of insurance, and seasonality of routes (Buixadé Farré et al. 2014; Gleb and Jin 2021). While global maritime trade occurs predominantly via the Panama and Suez Canals, the projections of sea ice reductions are likely to lead to increased accessibility and navigability of once impassable Arctic sea routes (Hauser et al. 2018; Mudryk et al. 2021). Furthermore, shipping routes through the Arctic are approximately 30% shorter than these alternatives (Smith and Stephenson 2013). Though unlikely to ever directly compete with the Suez or Panama Canals, developing the NSR further could lead to significantly reduced time and cost of Europe-to-Asia shipping during the ice-free summer and fall months (Liu and Kronbak 2010).

Although most Arctic shipping is destinational, the fact that the BSR is the only access point to the Arctic from the Pacific Ocean exposes it to numerous vessels passing through this region in route to Arctic ports. For example, a recent examination of voyages in the Bering Strait found that transient voyages (defined as voyages passing into and out of the region without stopping) made up nearly one third of all voyages between 2015 and 2020 (Kapsar 2022). Furthermore, the number of transient voyages increased by nearly 150% across the study period, with increases concentrated in Russian waters along the Northern Sea Route and in the Gulf of Anadyr. These findings indicate that vessels passing through the BSR are an emerging phenomenon that could pose increased environmental and safety risks to local communities and ecosystems without the possibility of concomitant economic benefits.

Currently, ecosystem impacts on the BSR are mitigated by sea ice preventing year-round shipping (Smith and Stephenson 2013). However, as sea ice extent continues to decrease and appear for shorter periods of time, there could be a greater impact on marine life and coastal communities (Herman-Mercer et al. 2016; Alaska Ocean Observing System and International Arctic Research Center 2021). At these current shipping levels, the effects are still being observed and felt by Indigenous communities and marine wildlife. If the NSR reached consistent commercial operation, it would pose serious threats to marine ecosystems and species in the Arctic (Yumashev et al. 2017; Hauser et al. 2018). More frequent shipping along the Northern Sea Route and the Northwest Passage will continue to generate increased vessel traffic throughout the BSR (AMSA 2009; Cao et al. 2022). As an emerging global gateway, it is important to prevent the exposure to further impacts that have transpired in other global gateways. These impacts include introduction of non-indigenous species, interaction of differing hydrographical temperature regimes that lead to trans-boundary movements of regional fish stocks, and shipping emissions, which contribute to climate change, generate acid rain and water contaminants, and negative impacts on human health (Golani 1998; Galil et al. 2015; El-Taybany et al. 2019). There are further policy opportunities in Arctic ocean planning to prioritize comprehensive and inclusive management practices to mitigate impacts, such as by using bridged value systems developed through Indigenous ocean planning or by integrating and expanding upon governance efforts such as the Northern Bering Sea Climate Resilience Area, established in 2016, that mandated the recognition and participation of Alaska Native Tribal governments during decision-making in this area (Raymond-Yakoubian and Daniel 2018).

Below, we discuss two unique subsets of vessel traffic in the BSR: natural resource development and cruise tourism. Each of these subsets has the potential to bring economic growth to the region, with associated socioeconomic and environmental effects.

### Natural resource development

Traffic through the Bering Strait has been impacted in recent years by Russia’s increasing operationalization of the NSR (Hartsig et al. 2012; Boylan 2021). Implementation of the Yamal LNG plant has promoted economic development in the Arctic Zone of Russia as well as increased shipping of LNG along the NSR (up to 16.5 million) (Rigot-Müller et al. 2022), which is expected to continue growing into the future (Katysheva 2019; Rigot-Müller et al. 2022). As of 2018, the Yamal LNG’s two operational production lines accounted for approximately 3.5% of the global LNG market (The Maritime Executive 2018). The production of the Yamal LNG is under long-term contracts in European and Asian markets and is expected to service markets year-round through its fleet of specifically designed ice-class LNG carriers that will transport product through the Bering Strait via the NSR in the summer (The Maritime Executive 2018). Therefore, there is a large potential for vessel traffic growth in the Yamal Peninsula, with potentially significant cascading spillover consequences for the Bering Strait Region (Rigot-Müller et al. 2022).

Beyond the Yamal Peninsula, expansion of oil and gas industries in the non-Russian Arctic and its subsequent effect on the BSR is uncertain compared to the expected impacts on the BSR from the growth in Russian Arctic oil and gas sectors. However, maritime vessel traffic in the BSR is projected to continue growing into the future (Huntington et al. 2020), as evidenced by the historical change in the number of transits through the Bering Strait, which increased ca. 2.5 times between 2008 and 2015 (from 220 to 540, respectively) (United States Coast Guard 2016). Much of this traffic growth was associated with oil and gas exploration off the northwest coast of Alaska.

The oil and gas industry requires the operation of several different types of vessels such as seismic survey vessels, drill ships, project cargo, heavy lift ships, ocean barges, construction vessels, and supply vessels (Hartsig et al. 2012). The growing presence of these different vessels also demonstrates that the expansion of oil and gas development constitutes a driver of the increase in vessel traffic.

Another natural resource development activity contributing to vessel traffic in the BSR is mineral development. Within the Arctic are large potential sources of minerals (e.g., phosphate, bauxite, iron) (Buixadé Farré et al. 2014). For example, one of the world’s largest zinc mines, Red Dog, is located off the northwest coast of Alaska in the northern part of the BSR. The mine has been producing zinc and lead powder concentrates since its inception in 1989 (Hasselbach et al. 2005; Due Kadenic 2015; Loeffler 2015; Neitlich et al. 2022). Zinc and lead are well-established industrial metals with critical applications in the global economy, such as for the galvanization of steel, creation of alloys, production of batteries, and other automotive demands (Mohr et al. 2018).

Though the mine has demonstrated some economic benefits to residents, it is important to recognize the negative impacts that such an extractive industry has on cultural traditions of Indigenous residents living in surrounding areas of the mine (Berman et al. 2020). Furthermore, associated environmental damages incurred by such mining operation over an extensive time should also be considered, including the dispersion of metal contaminants such as cadmium, lead, and zinc derived from the transportation of ore to the mine’s seaport (Hasselbach et al. 2005; Neitlich et al. 2022). This dispersion of contaminants incurs negative impacts on valuable tundra ecosystems (Hasselbach et al. 2005; Neitlich et al. 2022).

Other mining operations in the BSR include gold mining in Chukotka, one of Russia’s most productive gold regions, and near Nome, Alaska (Demuth 2019). The small-scale gold mining operations near Nome have gained popularity with reality TV appearing on Discovery Channel as *Bering Sea Gold* and *Gold Rush*. It demonstrates how another revenue outlet has been created by the historic and current development of mining activity in the BSR that results in cross-sector connections through distant interests such as television viewers. In addition, politics between Alaska and Russia in the early twentieth century were heavily influenced by the gold mining industry, reinforcing telecouplings (Demuth 2019). The global demands for minerals from these extractive industries render the BSR a telecoupled region, strengthened through the need from distant countries for these resources.

### Tourism

The telecoupled flow of tourism through and within the BSR is an area of current growth. Tourism can bring economic benefits to communities but can also leave ecological and social impacts (Liu and Lu 2014). In remote parts of the world, such as the BSR, tourism is also increasing the safety burden on communities, who are frequently the only search and rescue operators for hundreds of miles (Huntington et al. 2015). Cruise tourism within the BSR is a young and developing industry, with very limited information publicly available but with emerging issues (Fay and Karlsdóttir 2011; Cajaiba-Santana et al. 2020).

In other Arctic territories, the cruise ship industry is still a developing sector that is accompanied by steep travel expenses in often geographically secluded areas, which likely has contributed to a lack of research as a result (Marquez and Eagles 2007; Cajaiba-Santana et al. 2020). The Arctic regions that have received the most attention for cruise tourism related research and its effects are Arctic Canada, Svalbard, and Greenland (Ren et al. 2021). Much of this literature is concentrated on the impacts of cruise tourism development and the perceptions of local residents (Ren et al. 2021). Consensus in management is needed across the cruise ship industry in the Arctic as well as more incorporation of local perspectives in research (Marquez and Eagles 2007). Cruise tourism challenges vary widely among Arctic communities depending on political, social, and institutional structures and ability to respond to global changes (Ren et al. 2021).

The absence of assessment data of the tourism impacts causes difficulty when developing and implementing new policies that could potentially influence vessel traffic or conservation efforts (Cajaiba-Santana et al. 2020). For example, the Polar Code was implemented in 2017. However, there is no overarching governing body or authority to enforce the code or to monitor cruise shipping throughout the area (Cajaiba-Santana et al. 2020). Cruise ships are not adhering to established shipping routes for cargo vessels which complicates risk management (Silber et al. 2012). Thus, there is a need for greater government awareness and aid concerning these issues (Marquez and Eagles 2007). Evaluating how governance and policies shape the cruise ship industry will help demonstrate how the sector has developed over time and how it continues to impact the surrounding environment (Cajaiba-Santana et al. 2020).

Marine wildlife that migrates through the Bering Strait also contributed to the economies of distant regions by supporting the species these regions depend on for nature-based tourism. For instance, Eastern Pacific gray whales (*Eschrichtius robustus*) migrate through the BSR from their feeding grounds in the Arctic to breeding lagoons in Baja California Sur in northwestern Mexico (Dedina 2000). This brings in a significant number of tourists from around the world to Mexico to see this international whaling icon (Schwoerer et al. 2016). Mexico's reliance on tourism is evident by the number of tourists it receives (e.g., 29 million), tourism-related jobs it sustains (2 million), and the contributions to its coastal economies (Cisneros-Montemayor et al. 2020). The presence of gray whales contributes to coastal economies along the migratory route and whale watching destinations located in British Columbia, Oregon, California, and Mexico (Allen 2014; Schwoerer et al. 2016; Sullivan and Torres 2018). This example underlines how telecoupled connections in the BSR reach many distant systems and can generate multiple types of spillover effects.

## Spillover effects of telecoupling processes

Spillover effects are often missing or are being overlooked during traditional analyses. For example, traditional analyses on trade and payments for ecosystem services often focus on trade and payment partners (Liu and Yang 2013; Liu et al. 2018). Socioeconomic and environmental spillover effects are often recognized separately instead of simultaneously. In the context of global gateways, spillover effects of vessel traffic passing through the gateway include water contamination, non-indigenous species, and harmful algal blooms.

As larger vessels operate in and traverse through the BSR more frequently, the associated waste streams are a growing concern given the risks they generate to the environment (Parks et al. 2019). Waste streams from vessels may contain zoonotic pathogen-contaminated sewage, gray water, trash, oil, and other engine emissions. Below, we discuss waste streams associated with different vessel types as well as the potential effects of vessel waste on the BSR.

Different types of vessels are associated with different types and amounts of waste. For example, passenger ships carry a larger proportion of people compared to other vessel types, thus producing more sewage (e.g., drainage from toilet facilities or medical premises) and gray water (discharge from sinks, showers, laundry machines, and dishwashers). The US Clean Water Act mandates treatment of sewage before release into waters within 3 nautical miles of the shore (Parks et al. 2019). Sewage discharge poses various ecosystem risks such as fecal coliform, excess nutrient enrichment, and oxygen depletion. Although there are important measures in place to prevent the dumping of certain types of waste near ecologically sensitive areas (Parks et al. 2019), such as the Hanna Shoal (which lies on the outskirts of the BSR), monitoring and enforcement of these measures is challenging given the remoteness of these regions (Kuletz et al. 2015).

Another possible danger that water contamination poses is an increased prevalence of harmful algal blooms (HABs) and paralytic shellfish poisoning when coupled with increased water temperature (Parks et al. 2019). A study performed from 2012 to 2016 (excluding 2015) in the BSR discovered that 49% and 52% of walruses tested, had elevated levels of domoic acid and saxitoxin, respectively. These are neurotoxins that are released during HAB events (Quakenbush et al. 2016). Elevated toxin levels in marine mammals, such as walruses and seals, are a concern for local communities who rely on these resources for subsistence (Quakenbush et al. 2016; Larsen Tempel et al. 2021).

Along with the susceptibility of the BSR’s waters to HABs, its marine environment is becoming more suitable to the introduction of aquatic non-indigenous species via shipping (Droghini et al. 2020). The Bering Sea is suitable for growth and reproduction of all 42 assessed non-indigenous taxa from early July to mid-August with conditions suitable for 34 taxa year-round (Droghini et al. 2020). It is likely that the risk of non-indigenous species introduction will rise and compound with other stressors such as warming ocean waters to threaten the BSR’s ecosystem sustainability (Chan et al. 2019).

## Interaction of climate change and telecouplings in the Bering Strait Region

Historically, travel through the BSR has been seasonal with operations concentrated in summer months when the ice edge is north of the Bering Strait, and non-existent in the winter when ice-covered waters inhibit travel by most vessels which do not have ice-strengthening. In recent years, amplified Arctic warming has led to dramatic declines in the extent and duration of sea ice in the BSR (McCrystall et al. 2021). Decreased sea ice has resulted in a longer ice-free season and greater accessibility along the Northwest Passage and Northern Sea Routes (Pizzolato et al. 2016; Lynch et al. 2022). The increased accessibility of trade routes may thus further the vessel traffic (Mudryk et al. 2021). Additionally, changes in ecosystem function and patterns of marine mammal space use are expected due to loss of sea ice (Beatty et al. 2016). For instance, sea ice loss decreases the available habitat for ice-dependent marine mammals that rely on ice for reproduction, feeding, and shelter from predators (Kovacs et al. 2011).

Climate change may also indirectly affect the demand for fish products (i.e., by skewing demand toward products that are deemed more climate-friendly due to their harvesting method) (Troell et al. 2017). Climate change may increase the likelihood of conflict between fishery dependent nations as increased variability of ocean water temperatures affects the productivity and distribution of commercially harvested species (e.g., Atlantic mackerel conflict between the European Union, Norway, and Iceland) (Troell et al. 2017). The Central Arctic Ocean Fisheries Agreement (CAOFA) was implemented in 2018 in response to the evolving governance of a dynamic Central Arctic Ocean (CAO). This agreement intends to preemptively manage the inception of potential commercial fisheries in the CAO, as well as restrict unregulated fishing in the CAO, providing a basis for broader marine governance that focuses on the protection of Arctic marine resources, reduction of environmental impacts, and safety such as search and rescue to reduce the likelihood of conflict (Vylegzhanin et al. 2020).

Fisheries are a key resource that Arctic coastal communities—and countries around the world—rely on. Arctic fisheries contribute significantly to global marine aquaculture and are representative of large shares of gross domestic product (GDP) in several countries (e.g., 15% in Greenland, 10% in Iceland) (Troell et al. 2017). Therefore, fishery dependent countries are sensitive to changes in the abundance and availability of their fishing stocks and resources (Jansen et al. 2016). In Christiansen et al. (2014), the largest fisheries were bound to sub-Arctic/boreal waters such as the Bering Sea. Subsistence fisheries catches in the Arctic seas also accounted for 950,000 tons from 1950 to 2006 (Christiansen et al. 2014). Fisheries illustrate important relationships between global resource and demand systems linking distant processes around the world, further investigated at a regional scale such as in the BSR (Troell et al. 2017). A summary of potential spillover effects can be found in Table 2. When combined with increased interest in the Arctic’s natural resources in recent decades, these changes have raised the number and strength of distant connections between Arctic and non-Arctic CHANS.

**Table 2:** Summary of potential telecoupling spillover effects impacting the Bering Strait. [1] (Wang et al. 2020) [2] (Mudryk et al. 2021) [3] (AMSA 2009) [4] (Parks et al. 2019) [5] (Huntington et al. 2015) [6] (Quakenbush et al. 2016) [7] (Kaiser et al. 2021) [8] (Sheffield et al. 2021) [9] (Tolvanen et al. 2019) [10] (Due Kadenic 2015) [11] (Gadamus and Raymond-Yakoubian 2015) [12] (Gewin 2019) [13] (Vierros et al. 2020) [14] (Xiong et al. 2018)

Telecoupling	Effects
Tourism	Infrastructure development [1]
	Increase in tourism-related jobs [1]
	Increased greenhouse gas emissions [2]
	Expanded travel seasons and activity areas [1, 3]
	Waste streams [4]
Shipping	Increased risk of ship strikes, oil spills, and noise pollution [5]
	Increased greenhouse gas and black carbon emissions [2]
	Waste streams [4]
	Harmful algal blooms [4, 6]
	Overexploitation of fisheries [7]
	Garbage pollution and foreign marine debris [8]
Resource development	Formation of collaboration communities [9]
	Employment opportunities [10]
	Acid mine drainage and runoff [9]
	Deposition of submarine tailings [9]
	Oil spills [5]
	Heavy metal dispersal [9]
	Interference with Indigenous rights and sovereignty [11, 12, 13]

## Distant geopolitical systems and Arctic interests

Much of the expected traffic throughout the Northern Sea Route passes through the Russian side and utilizes Russian ports where US laws and policies have minimal or no influence. As Russia’s actions and relations with Western countries continue to be strained, Russian leaders maintain an interest in diversifying the country’s influence on other regions, specifically in the Arctic (Boylan 2021). For instance, Russia continues to be a global leader in hydrocarbon production, with 83% and 12% of its gas and oil production, respectively, occurring in the Arctic zone (Kirsanova et al. 2020). Furthermore, Russia has fostered oceanographic research to defend its claims to the continental shelf in the Arctic Ocean (Boylan 2021). However, military conflict may affect Russia’s natural resource exploitation in offshore oil fields, which so far has occurred with the aid of foreign partners (Kaczynski 2013; Tillman et al. 2018).

Not only is the Arctic attracting the interest of Arctic states, but it is also attracting the interest of non-Arctic countries such as China, Japan, and India (Lajeunesse 2018; Tillman et al. 2018; Kirsanova et al. 2020). Due to China’s large dependence on Russian, African, and Middle Eastern oil, China has exhibited growing interests in the Arctic (Buixadé Farré et al. 2014; Tillman et al. 2018; Wang et al. 2020). Since 80% of China’s oil imports originate from the Middle East and Africa and must pass through the Strait of Malacca and the South China Sea, this exposes China to an overdependence on unstable sources and potentially hostile maritime neighbors (Buixadé Farré et al. 2014; Wang et al. 2020). Therefore, China’s approach to exploiting the Northeast Passage (which shares significant overlap with the Northern Sea Route) for its energy resources, makes China a prime candidate for bolstering relations with Russia. In July of 2017, China announced plans to collaborate with Russia on Arctic infrastructure to build a “Polar Silk Route (PSR)” (Zhang et al. 2019). In January 2018, China released its first white paper on Arctic policy expanding on their vision for the PSR and international governance and collaboration (Tillman et al. 2018). While much of China’s LNG sources are transported via pipelines overland from Russia, China will benefit from the diversification of shipping import routes and suppliers for LNG to accompany overland pipelines. This can reduce the impact of shocks to their primary transportation routes such as the Strait of Malacca, especially as the transition from coal-to-gas accelerates in the future (Yin and Lam 2022).

## Governance

Geographically, there are two countries in the BSR (US and Russia); however, the BSR—like many other Arctic regions—is a geopolitically diverse and multi-jurisdictional area due to its international waters and classification as an international strait under the United Nations Convention on the Law of the Sea (UNCLOS) (Huntington et al. 2015). This convention mandates that passage through the Bering Strait must be unimpeded for all traversing vessels (Huntington et al. 2015). Furthermore, Arctic regions are often influenced by the geopolitical actions of non-Arctic countries as the Arctic continues to develop (Moe and Stokke 2019). The development of shipping routes connecting southern regions to the Arctic waters, infrastructure investments, and diplomatic initiatives that strengthen ties between Arctic and non-Arctic states (e.g., the PSR (Zhang et al. 2019)) are all a result of telecoupling processes that are invoking the transition into a globally inclusive Arctic future (Paglia 2018).

Additionally, the vulnerability of Arctic marine systems is dependent on governance and policy that plays an important role in managing shipping and tourism in the region. Thus, the BSR’s environmental security is dependent on political stability and sensible stewardship of its marine resources as it undergoes many dynamic environmental changes (Berkman et al. 2016).

National governments are major decision makers that have enormous influence over the region’s activities. The United States and Russia have participated in several environmental and maritime agreements that apply to the BSR. The two nations also involve the International Maritime Organization (IMO) when creating regulations regarding navigational safety and pollution prevention since the United Nations agency responsible for the establishment of guidelines for the operation of ships in polar waters (Berkman et al. 2016). The IMO’s Marine Environment Protection Committee also has several projects and guidelines designed to help combat environmental concerns, such as non-indigenous species. However, the IMO is a regulatory organization, and it is up to its member states to apply and enforce its guidelines and regulations.

Other international agreements pertaining to the BSR include the UNCLOS, Convention on International Trade in Endangered Species of Wild Fauna and Flora (CITES), and the International Convention for Safety of Life at Sea (SOLAS). The Arctic Council, an intergovernmental forum that fuses the cooperation of the eight Arctic states (Canada, Denmark (Greenland), Finland, Iceland, Norway, Russia, Sweden, and the United States), also has several initiatives that are applied across the regions. They include the Arctic Monitoring and Assessment Programme, Conservation of Arctic Flora and Fauna, Protection of the Arctic Marine Environment, and Emergency Prevention, Preparedness and Response. There are also many regional and bilateral agreements in effect between the US and Russia (Berkman et al. 2016). The US federal government also coordinates monitoring and observations of Arctic Alaska through the Interagency Arctic Research Policy Committee, the Study of Environmental Arctic Change Program, and the Arctic Research Consortium of the US (de la Barre et al. 2016).

The US and Russian Coast Guards help implement certain policies made by other agents or decision-making entities (Berkman et al. 2016). For example, the Agreement to Combat Illegal, Unreported, and Unregulated (IUU) Fishing guides their cooperation to adequately manage marine sustainability issues such as illegal fishing, specifically relating to the crab stocks of the Bering Sea (Berkman et al. 2016). The governance of the BSR is most effective when involved agents maintain open communication and cooperation to ensure alignment with the overarching laws of the sea and international agreements (de la Barre et al. 2016). Following Russia’s invasion of Ukraine, collaborations with Russia have likely ceased for the foreseeable future. For instance, the Arctic Council has announced they are ‘pausing’ their collaborative work due to Russia’s actions against Ukraine (Schreiber 2022). Uncertainty surrounding the effective management, data sharing, and research collaborations between nations involving the Arctic increases when there is geopolitical conflict, such as the Russia-Ukraine war, that has effectively paused collaborative work by the Arctic Council (Goodman and Wieffering 2022; Holdren et al. 2022).

Arguably, as many of the residents of the BSR are Indigenous Peoples, Indigenous community members are the most directly impacted by the effects and regulations concerning telecoupling processes in the BSR. The scourge of colonialism has deeply afflicted Indigenous communities, and there have been many challenges between Alaskan tribes and the state government of Alaska (Carothers et al. 2021). Decisions made by the federal and state governments affect these tribal communities (Carmack et al. 2012). In return, these communities lobby for their rights and sovereignty to have equitable access and representation during decision-making in a court of law (Gadamus and Raymond-Yakoubian 2015; Gewin 2019; Vierros et al. 2020). The International Whaling Commission (IWC) provides a means for residents to have a voice internationally. Partnerships between Indigenous Peoples and other governing institutions are essential for ensuring the sustainability and resilience of the BSR and subsistence into the future (Siders et al. 2016; Hauser et al. 2018). To move forward toward a more equitable and inclusive future both in government and environmentally (Raymond-Yakoubian and Daniel 2018; Liu et al. 2021), the adversity of Indigenous communities with Western government must not be erased but rather reflected on to acknowledge and raise the voices of those directly affected today.

Currently, there are many bilateral and international agreements between agents in the BSR. These agreements have varying purposes, whether it is to deter pollution, increase navigational safety, protect threatened or endangered species, or involve Indigenous views and management methods (Berkman et al. 2016). However, Indigenous voices and traditional knowledge are still largely lacking from the creation of many of these policies (e.g., governance of the global ocean commons (Vierros et al. 2020)) often in part due to a lack of analogous Indigenous departments or agencies that manage natural resources (e.g., National Oceanic and Atmospheric Administration) which hinders their capacity to actively participate (Raymond-Yakoubian and Daniel 2018). This must change to create effective and inclusive policies that promote the safety and sustainability of traditional subsistence livelihoods and minimize vessel-wildlife conflict (Raymond-Yakoubian and Daniel 2018).

When it comes to governance and management of telecoupling, there are distinct perspectives to remember (Newig et al. 2019). Applying this to the BSR, governance can either induce telecoupling (e.g., development of natural resource industries and global trade in the BSR), coordinate telecoupled flows (e.g., formulate commodity chains due to industry development), or respond to telecoupling and its negative external factors (e.g., sustainability issues generated in spillover regions by the industry expansion). These perspectives often occur sequentially during a telecoupling process, but this may not always be the case (Newig et al. 2019).

Governance in the BSR should be based on making collaborative decisions through the analysis of environmental, social, and economic impacts that cause or result from these decisions. Telecoupling relationships that are also applicable to other global gateway CHANS can be more readily recognized by prioritizing the BSR’s biodiversity and local communities during these decisions. The international and multi-jurisdictional nature of the BSR may cause difficulties when recognizing effects due to telecoupling processes, especially as a spillover region. Consequently, telecouplings must first be explicitly accounted for and understood sufficiently before being addressed by governance. The effects of anthropogenic stressors such as climate change and human activities like shipping, tourism, and resource development are felt in other global gateways around the world. However, challenges remain with allocating mitigation responsibility in response to telecoupling processes, such as shipping, that have far-reaching climate and health impacts (Liu et al. 2019). Allocation of responsibility requires quantitative analysis when examining relationships between the global economy, shipping, and ecological connectivity (Liu et al. 2019). Therefore, investigating the BSR’s telecoupling dynamics and spillover systems, both qualitatively and quantitatively, is useful for identifying changes in global gateways at the local scale.

## Future research directions

Framing the BSR through an interdisciplinary lens such as the telecoupling framework can advance the knowledge of and methods used to analyze global gateways. However, there are many remaining problems to be addressed concerning the BSR. Arctic sea routes face a multitude of challenges such as costly management and development, geopolitical tensions, harsh climate conditions, jurisdictional conflicts, lack of infrastructure (e.g., deepwater ports), limited search and rescue operations, and shallow waters (Buixadé Farré et al. 2014). Feasibility, logistics, and profitability of Arctic sea routes and their effects on global shipping networks requires the assessment of environmental parameters, policy barriers, and shipping aspects (e.g., load rate, ice-strengthened capability, bunker prices) (Milaković et al. 2018; Guo et al. 2022). For example, the analysis of fully operational Arctic sea routes has been applied to examine how China–Europe shipping routes will be reshaped and either strengthened or weakened (Guo et al. 2022). This research can be further expanded to encompass how the intensity of shipping using Arctic sea routes through the Bering Strait will amplify the impacts in this critical bottleneck, as it develops into a global gateway. Continuing research on the regional differences between other global gateways and their economies and shipping frequencies will encourage development of important theoretical advances and practical applications.

The BSR has a significant number of Indigenous communities, with a large proportion of people dependent on subsistence harvesting activities (Hartsig et al. 2012; Larsen Tempel et al. 2021; Todorov 2022). These communities experience the effects of shipping, natural resource development, and tourism directly. Ensuring balance between managing the livelihoods of Indigenous communities, sustainability of marine ecosystems, and economic development creates complex layers to governance and policy. Investigating traditional management methods by Indigenous communities can help build equitable and fair policy with respect to Indigenous culture and the fragile biodiversity of the BSR (Gadamus and Raymond-Yakoubian 2015).

Examining how telecouplings originate and evolve will create a basis for recognizing their effects on the BSR, its communities, and marine systems (Sala et al. 2021). Ecosystem services like those provided by marine systems would be more effectively managed using information from comprehensive studies that encompass the impacts from multiple, interacting telecouplings (Liu et al. 2015; Ouyang et al. 2020). New interdisciplinary approaches are needed to address these impacts and are necessary when creating mitigation strategies and policies (Platjouw and Soininen 2019) in telecoupled systems.

While research at broad geographic scales (e.g., national, continental, global) provide a general understanding of telecoupling dynamics, regional and local scales should also be explored. This approach can foster explicit insights into the dynamics of telecoupled systems and expand research into agents, flows, and spillover effects (Liu and Yang 2013). Due to the global nature of telecouplings such as shipping, information gained from studies at local and regional scales can be tailored to apply in different gateways worldwide. Therefore, examining global gateways as telecoupled human and natural systems that vary in geographic size, population, biodiversity, and physical structure is essential for synthesizing information about telecoupling dynamics at different scales.

## Conclusions

The BSR provides a unique opportunity to investigate global gateways as telecoupled human and natural systems. Despite published research focusing on the effects of individual telecouplings, there is little research concerning their interrelationships and how spillover effects from multiple telecouplings affect vessel traffic (Arctic Council 2016; Herzberger et al. 2019). The flows within this region shape the Arctic into an area that is conceptualized using its human connections across space rather than just a geographic region (Paglia 2018). The BSR’s role as a bottleneck and key migratory corridor makes it a model system for studying telecouplings such as shipping, tourism, and resource development, which are not unique to this region and occur worldwide. The BSR’s system and interconnections allow the investigation of CHANS dynamics in an area where there is high risk for potentially negative environmental outcomes, while also allowing the development and implementation of mitigating actions. The unifying framework and terminology used here may contribute to the synthesis of related research in the Bering Strait and similar shipping corridors. Analyzing global gateways as telecoupled human and natural systems provides a foundation for a more comprehensive understanding of complex human-nature interactions. This understanding will also support governance that focuses on long-term ecosystem protection and societal development, while accommodating global environment and social change in an increasingly interconnected world (Croissant and Pelke 2022; Viña and Liu 2022).
